# *Premna vietnamensis* (Lamiaceae, Premnoideae), a distinct new species from the Central Highlands of Vietnam

**DOI:** 10.1371/journal.pone.0195811

**Published:** 2018-05-02

**Authors:** Do Van Hai, Dao-Zhang Min, Nguyen Sinh Khang, Yun-Hong Tan, Pham Thi Kim Thoa, Gemma L. C. Bramley, Rogier P. J. de Kok, Bo Li

**Affiliations:** 1 Institute of Ecology and Biological Resources, Vietnam Academy of Science & Technology, Cau Giay, Hanoi, Vietnam; 2 College of Agronomy, Jiangxi Agricultural University, Nanchang, Jiangxi, China; 3 Southeast Asia Biodiversity Research Institute, Chinese Academy of Sciences, Yezin, Nay Pyi Taw, Myanmar; 4 Center for Integrative Conservation, Xishuangbanna Tropical Botanical Garden, Chinese Academy of Sciences, Mengla, Yunnan, China; 5 Faculty of Environment, Danang University of Science and Technology, Danang, Vietnam; 6 Herbarium, Royal Botanic Gardens Kew, Richmond, Surrey, United Kingdom; 7 Singapore Botanic Gardens, National Parks Board, Singapore, Singapore; Missouri Botanical Garden, UNITED STATES

## Abstract

*Premna vietnamensis*, a distinct new species which is endemic to Gia Lai Province in Central Highlands of Vietnam, is described and illustrated. It is characterized by its calyx tube bearing a semi-globose fleshy appendage, which has not been reported before from all known congeneric taxa, as well as from the Lamiaceae. A phylogenetic analysis of the whole Lamiaceae based on a sampling including representatives from all 12 currently recognized subfamilies confirmed the placement of this new species within *Premna* of the Premnoideae. Morphologically and geographically, *P*. *vietnamensis* is most similar to *P*. *stenobotrys*, but differs significantly in many aspects.

## Introduction

The genus *Premna* L. was traditionally placed in the subfamily Viticoideae Briq. of Verbenaceae [1,2], but is now transferred to the subfamily Premnoideae Bo Li, R.G. Olmstead & P.D. Cantino in Lamiaceae [3]. With more than 100 species recognized (WCSP, http://wcsp.science.kew.org), *Premna* is one of the largest woody genera in the mint family, and mainly distributed in Old World tropical and subtropical regions from Africa to China, throughout southern Asia, to northern Australia, and various islands in the Pacific and Indian Oceans [4,5]. In the past 30 years, a number of regional revisions of the genus have been published, such as for Australia [6], China [2], India [7], Malaysia [5,8], New Caledonia [9], Sri Lanka [10], Thailand [11], and Vietnam [12, 13], and many new taxa were discovered and described [5,14–20].

**Fig 1:**
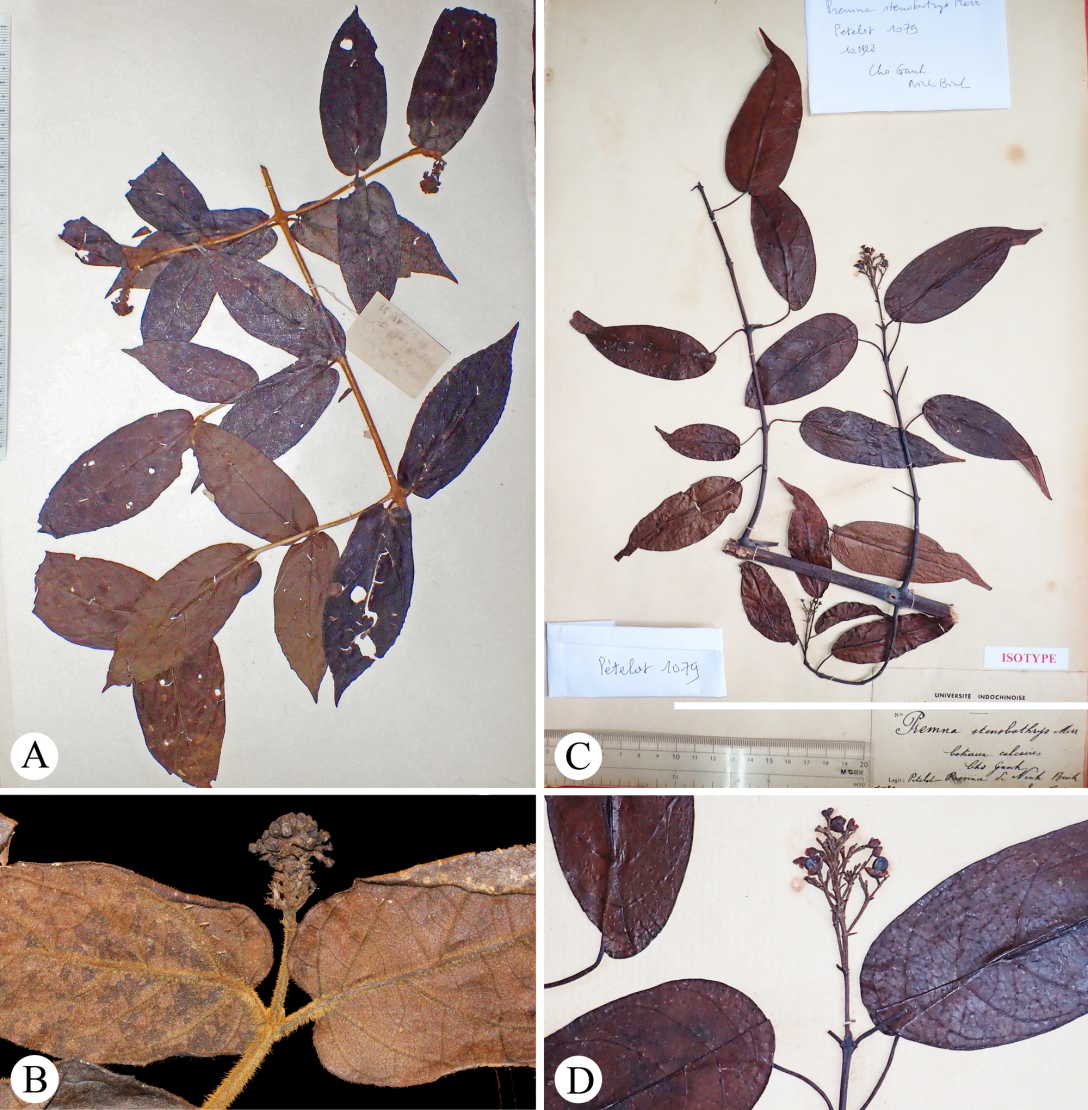
Specimens of *Premna vietnamensis* (A, B) and *P*. *stenobotrys* (C, D). **A,** Branchlets, leaves and inflorescences of *P*. *vietnamensis* taken from *L*.*K*. *Bien 830* (HN). **B,** An inflorescence of *P*. *vietnamensis* taken from *VK 4457* (HN) showing dense golden hairs on branchlets, petioles, and peduncles. **C,** Isotype of *P*. *stenobotrys*, *P*.*A*.*Pételot 1179* (HNU). D, An infructescence of *P*. *stenobotrys* taken from *P*.*A*.*Pételot 1179* (HNU).

**Fig 2:**
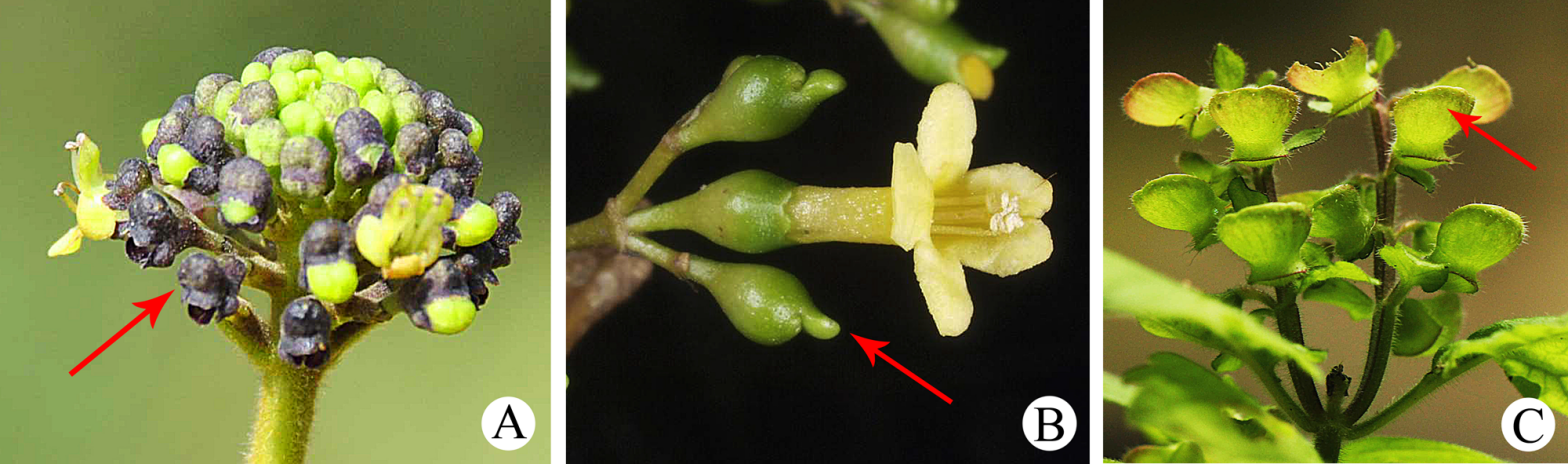
Comparisons of calyx morphology of *Premna vietnamensis* (A), *Vitex gamosepala* (B), and *Scutellaria* L. (C). **A,** An inflorescence of *P*. *vietnamensis* showing the semi-globose fleshy appendage on calyx tube (indicated by the arrow). **B,** A cyme of *V*. *gamosepala* showing the fleshy calyx lob (indicated by the arrow). **C,** An infructescence of *S*. *indica* L. showing the typical dorsal shield on the calyx of *Scutellaria* species (indicated by the arrow).

During an academic visit to Vietnam in 2016, Dr. Bo Li examined *Premna* specimens deposited in the herbarium of the Institute of Ecology and Biological Resources of the Vietnam Academy of Science and Technology (HN) in Hanoi and sorted out several unidentified specimens from those of *P*. *stenobotrys* Merr. These specimens were all collected from K’Bang District of Gia Lai Province, one of the five provinces in the Central Highlands of Vietnam, and had been collected between 1978 and 2011 (see Fig 1A and 1B for examples). Although the collections were determined to be morphologically identical to each other, they did not match any species currently recognized in *Premna*. Superficially, the unidentified taxon resembles *P*. *stenobotrys* (Fig 1C and 1D), but apparently differs in an indumentum of dense golden hairs throughout. After careful dissection of few flowers taken from *L*.*K*. *Bien 830* (HN) and examination of digital vouchers of *VK 4457* (HN) provided by Dr. Do Van Hai, we found that this plant bears an atypical structure on its calyx tube: a semi-globose fleshy appendage (Fig 2A). After consulting several Lamiaceae experts and a few taxonomists who are familiar with the flora of tropical Asia, we were told that such a calyx structure has not been observed in Lamiaceae, and its corolla is not similar to any known *Premna* species. In Lamiaceae, a slightly similar calyx appendage can be found in *Vitex gamosepala* Griff. of the subfamily Viticoideae (Fig 2B) or an outgrowth on the calyx forming a shield is common in *Scutellaria* L. species of the subfamily Scutellarioideae (Fig 2C), but the structure of these differs from that of the unidentified taxon significantly. In the absence of any mature fruits or material suitable for DNA extraction, further analysis was not possible. To solve this problem, in April 2017, living plants and mature fruits of the plant were recollected successfully in Gia Lai. This provided an opportunity to infer the phylogenetic position of the new taxon in the context of the phylogeny of the Lamiaceae. Finally, its membership within *Premna* was supported by both morphological and molecular characters, and we can confirm that it represents a hitherto undescribed species after comparing the plant with congeneric taxa. It is named as *Premna vietnamensis*, and here reported.

## Materials and methods

### Ethics statements

Collection of this new species was conducted in compliance with existing regulations for plants defined as non-commercial, as determined by local government offices. Our field investigations did not take place in any natural conservation area and no specific permissions were required for visiting the location of the new species. In addition, our field studies did not involve any endangered or protected species.

### Nomenclature

The electronic version of this article in Portable Document Format (PDF) in a work with an ISSN or ISBN will represent a published work according to the International Code of Nomenclature for Algae, Fungi, and Plants [21], and hence the new names contained in the electronic publication of a PLOS article are effectively published under that Code from the electronic edition alone, so there is no longer any need to provide printed copies.

In addition, the new name contained in this work has been submitted to IPNI, from where it will be made available to the Global Names Index. The IPNI LSID can be resolved and the associated information viewed through any standard web browser by appending the LSID contained in this publication to the prefix http://ipni.org/. The online version of this work is archived and available from the following digital repositories: PubMed Central and LOCKSS.

### Phylogenetic analysis

#### Taxon sampling and molecular data set

In order to infer the subfamilial placement of the new species in Lamiaceae, chloroplast DNA sequences *matK*, *rbcL*, and *trnL-F* were generated from its three representatives, and added to a combined data set which was simplified from the data set D155 in Li et al. [3]. The final data set included 64 ingroup accessions in Lamiaceae covering all 12 currently recognized subfamilies [3,4,22] and five outgroup accessions representing Mazaceae, Paulowniaceae, Phrymaceae, and Orobanchaceae which are the closest relatives to Lamiaceae in Lamiales [23,24]. The voucher information and GenBank accession numbers are provided in S1 Table.

#### DNA extraction, amplification and sequencing

Total genomic DNA was extracted by using DNEasy^®^ Plant Mini Kit (QIAGEN^®^, Valencia, California, USA) following the manufacturer’s specifications. The DNA extracts were dissolved in TE buffer and preserved at −20°C for further use. A single PCR Polymerase chain reaction (PCR) system and protocol was used for amplification of the three chloroplast regions. Primer pairs, PCR reaction, and amplification program followed Li et al. [3]. PCR products were visualized using agarose gel electrophoresis and sequencing was served by the Sangon Biotech (Shanghai) Co., Ltd., using the same primers for PCR amplifications.

#### Alignment and phylogenetic analyses

Sequences were assembled and edited using Sequencher v.4.5 (Gene Codes Corporation, Ann Arbor, Michigan, U.S.A.). Sequence alignment was initially conducted in Clustal X v.2.0. [25] and adjusted manually in BioEdit Sequence Alignment Editor v.7.0.0 [26]. The combined data set was analyzed using ML and Bayesian inference (BI) methods with gaps treated as simple indels determined by the program Gapcoder [27].

ML analyses were performed on the web server RAxML Black Box [28]. Before each submission, the “Maximum likelihood search” and “Estimate proportion of invariable sites” options were selected, with a total of 1000 bootstrap replicates performed.

BI analysis was executed using MrBayes version 3.2.2 [29]. The best substitution types (Nst) and rate distribution models (rates) were determined by the Akaike information criterion (AIC) using Model Test v.3.7 [30] with the hierarchical likelihood ratio tests. Four Markov chain Monte Carlo (MCMC) chains were run, each beginning with a random tree and sampling one tree every 100 generations for 20 000 000 generations. Convergence was assessed using the standard deviation of split frequencies, with values *<* 0.01 interpreted as indicating good convergence. The first 25% of samples were discarded as burn-in, and the post-burn-in samples summarized as a 50% majority-rule consensus tree.

### Morphological observations

The morphological description of the new species was based on observation and measurement of fresh and dried specimens, as well as materials preserved in FAA solution (formalin: acetic acid: alcohol = 18: 1: 1). Morphological variation was measured using a ruler and a micrometer. Morphological comparisons within *Premna* were based on specimens or their digital photos held at A, BM, CDBI, E, HN, HNU, IBSC, K, KUN, L, MO, NAS, NY, P, PE, S, US and WH (institutional acronyms follow the Index Herbarium [31]), and on field photographs of some Asian species. High resolution images of the type specimens of *P*. *stenobotrys* (held at A, barcode nos. A00099807, A00099808) were consulted on JSTOR Global Plants (http://about.jstor.org/, accessed 10 October 2017), and one of the isotypes deposited at HNU was examined under a stereo dissecting microscope.

## Results and discussion

### Phylogenetic position of the new species

**Fig 3:**
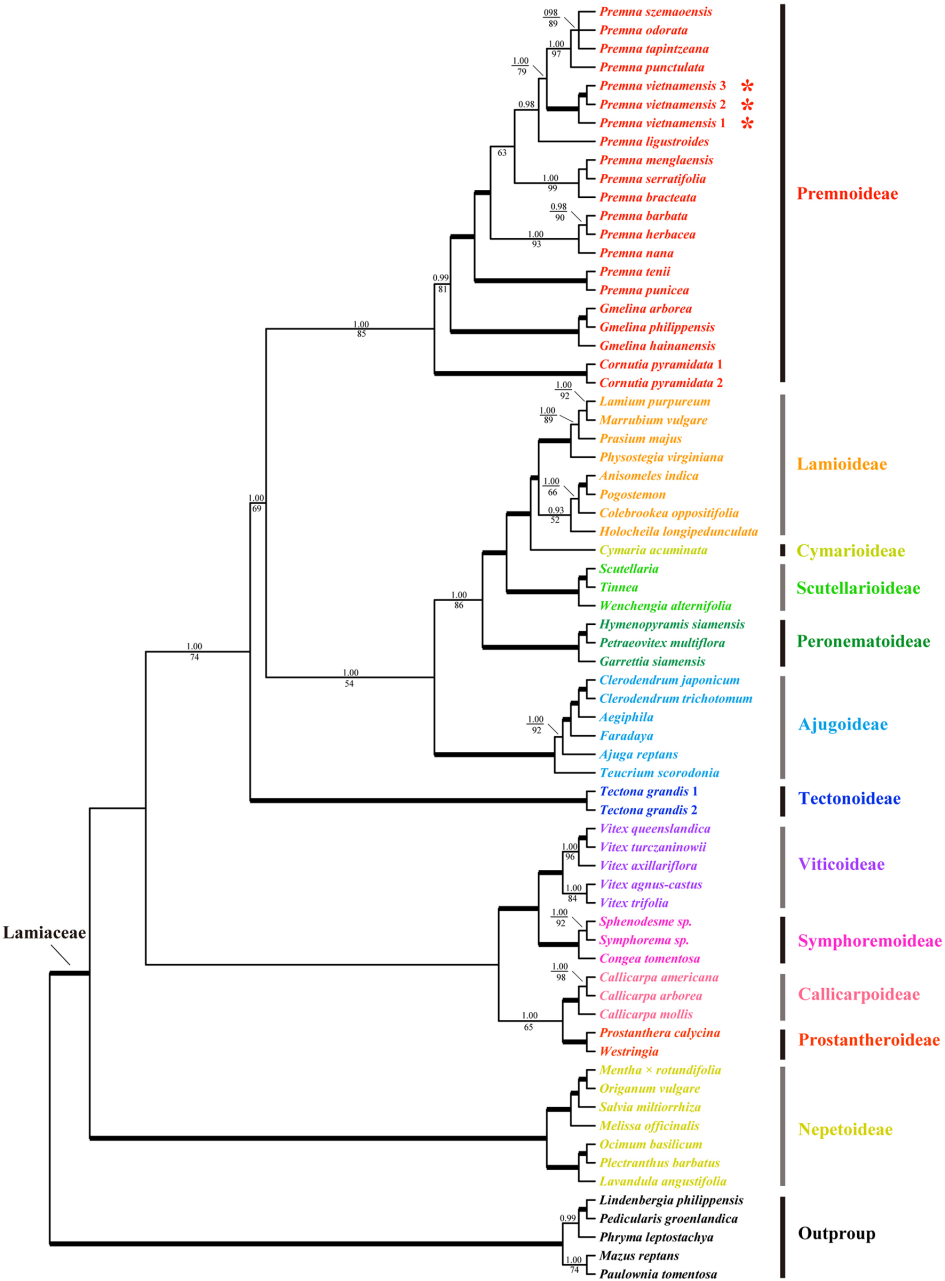
Bayesian 50% majority-rule consensus tree of Lamiaceae based on the combined cpDNA (*matK* + *rbcL* + *trnL-F*) data set, with gaps treated as simple indels. The topology of the ML tree is congruent with the BI tree. The 12 subfamilies of Lamiaceae are marked by different colors. Numbers above and below the branches are Bayesian posterior probabilities (PP) and maximum parsimony bootstrap values (BS), respectively. The bold lines indicate the PP = 1.00 and simultaneously BS = 100. The absent numbers mean the support value ≤ 65% for BS or 90% for PP. The symbol “*” marks the phylogenetic position of *P*. *vietnamensis* represented by three accessions. A single generic name indicates that the combined sequences were pooled from different species of the genus. Multiple accessions of the same species are numbered according to S1 Table.

Based on the combined data set of three chloroplast DNA sequences (*matK*, *rbcL*, and *trnL-F*), BI and ML analyses yielded highly congruent topologies, thus the 50% majority-rule consensus tree from the BI analysis was here presented (Fig 3). Twelve highly supported clades were obtained, and correspond to the 12 subfamilies recognized in Li et al. [3], Harley et al. [4], and Li & Olmstead [22], viz., Ajugoideae, Callicarpoideae, Cymarioideae, Lamioideae, Nepetoideae, Peronematoideae, Premnoideae, Prostantheroideae, Scutellarioideae, Symphorematoideae, Tectonoideae and Viticoideae. Relationships among the subfamilies are mostly consistent with those presented in Li et al. [3]. Significantly, the three representatives of *P*. *vietnamensis* formed a highly supported clade (posterior probability, PP = 1.00, ML bootstrap, BS = 100) and was deeply nested within the genus *Premna* which received moderate to high supports (PP = 1.00, BS = 85). Although the placement of the new species within *Premna* was confirmed, it is not possible to infer the phylogenetically closest species to *P*. *vietnamensis* because the sample of *Premna* included was not representative of the species diversity within the genus.

### Morphological comparisons

In *Premna*, *P*. *vietnamensis* is morphologically and geographically most similar to *P*. *stenobotrys*. They are both woody climbers having ovate-oblong to elliptic leaves and congested thyrses, and their inflorescences extend as they become infructescences. *Premna stenobotrys* occurs in a similar habitat as *P*. *vietnamensis*, frequently climbing on many kinds of shrubs along roadside and evergreen broad-leaved forest edges. Furthermore, though the type specimens of *P*. *stenobotrys* were collected from northern Vietnam, it is widely distributed from central to northern regions of the country based on specimen records, as well as our own field observations, and it was also reported from Thailand [32]. However, *P*. *vietnamensis* can be immediately distinguished from *P*. *stenobotrys* by its indumentum of dense, spreading, golden-brown hairs throughout (vs. nearly glabrous or easily glabrescent), significantly shorter petioles (2.0–6.0 mm vs. 15–50 cm), greenish yellow corolla (vs. red), and exserted stamens (vs. included). The indumentum, corolla and stamens of *P*. *vietnamensis* particularly resemble *P*. *fulva* Craib, a species widely distributed in Thailand, Laos, Vietnam and southwest China [33], but *P*. *fulva* differs from *P*. *vietnamensis* in having ovate to suborbicular leaves and flat-topped corymboses. Additionally, the fruits of *P*. *vietnamensis* are very similar to those of *P*. *clavata* de Kok [5], a newly described and remarkable species from Malaysia. The endocarp of both species is noticeably warty, and their fruits have only one well developed seed. On the basis of this fruit type, *P*. *vietnamensis* obviously belongs to the “*P*. *trichostoma*-group” [34], but apparently differs from every species in the same group.

Based on the above molecular and morphological evidences, we can confirm that the plant discussed here represents a hitherto undescribed species, thus formally describe it as below.

### Taxonomic treatment

**Fig 4:**
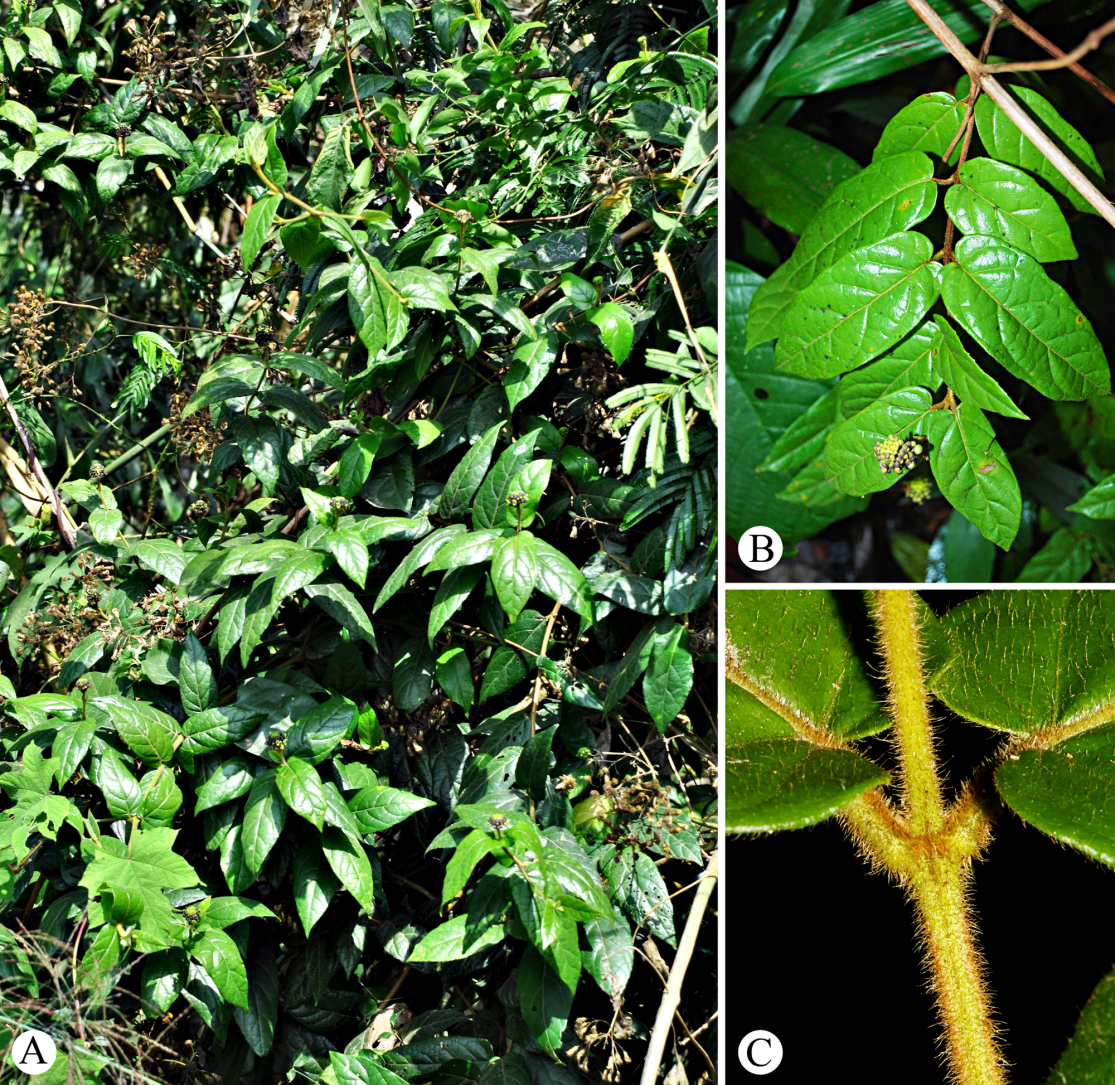
Premna vietnamensis. **A**, Habitat and habit. **B**, A branch and a branchlet with inflorescence. **C**, Leaf bases and petioles.

**Fig 5:**
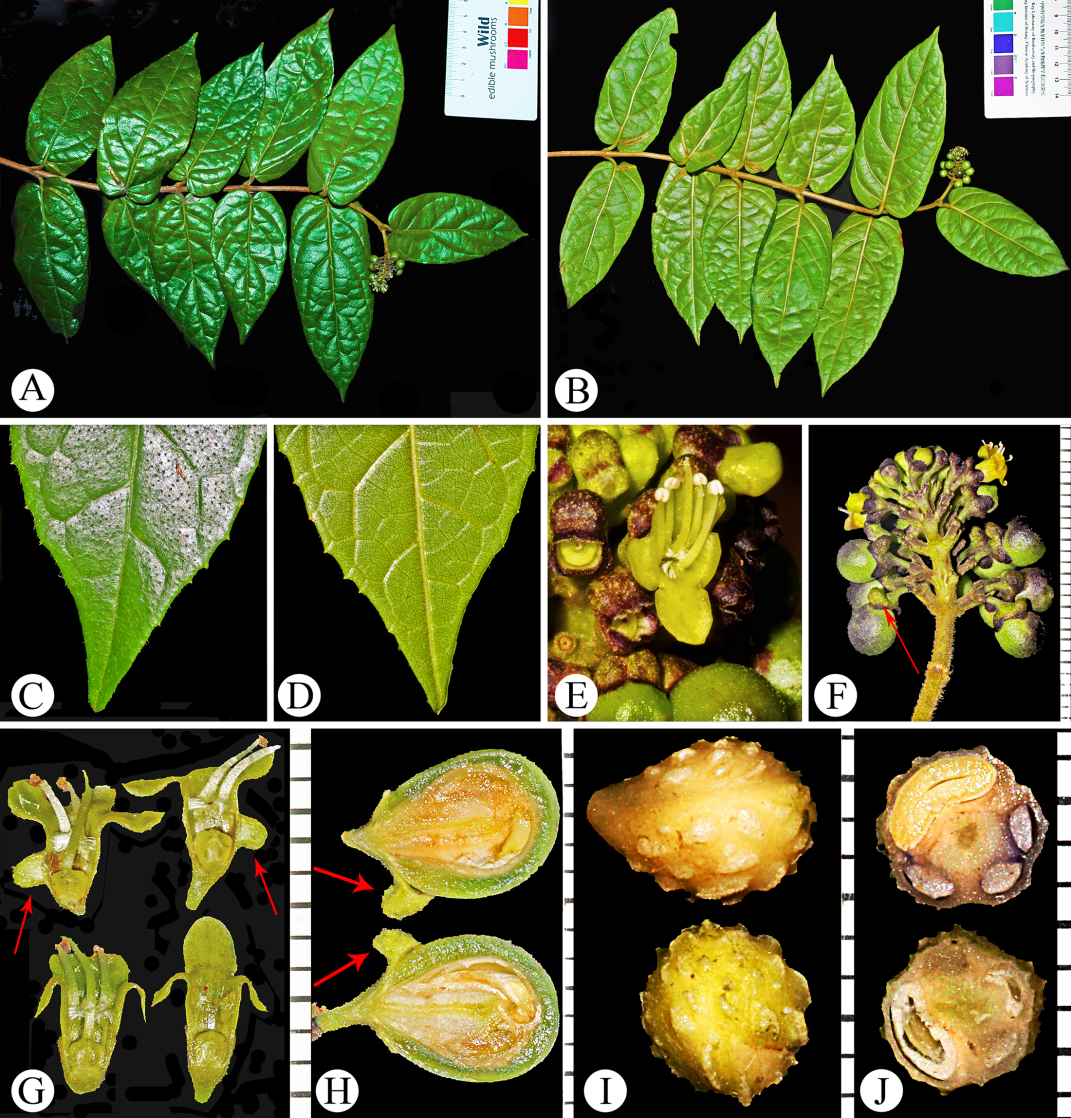
Morphology and anatomy of *Premna vietnamensis*. **A**, Adaxial view of a fruiting branchlet. **B**, Abaxial view of a fruiting branchlet. **C**, Adaxial view of a leaf apex. **D**, Abaxial view of a leaf apex. **E**, Flower. **F**, Infructescence and mature fruits with persistent calyces. **G**, Radial (upper) and tangential (lower) dissections of flowers. **H**, Vertical dissections of a fruit. **I**, Equatorial (upper) and polar (lower) view of a pyrene. **J**, A cross section of a pyrene. All arrows pointed to the fleshy appendage on calyx tube. Ruler scale in F–J is 1 mm.

**Fig 6:**
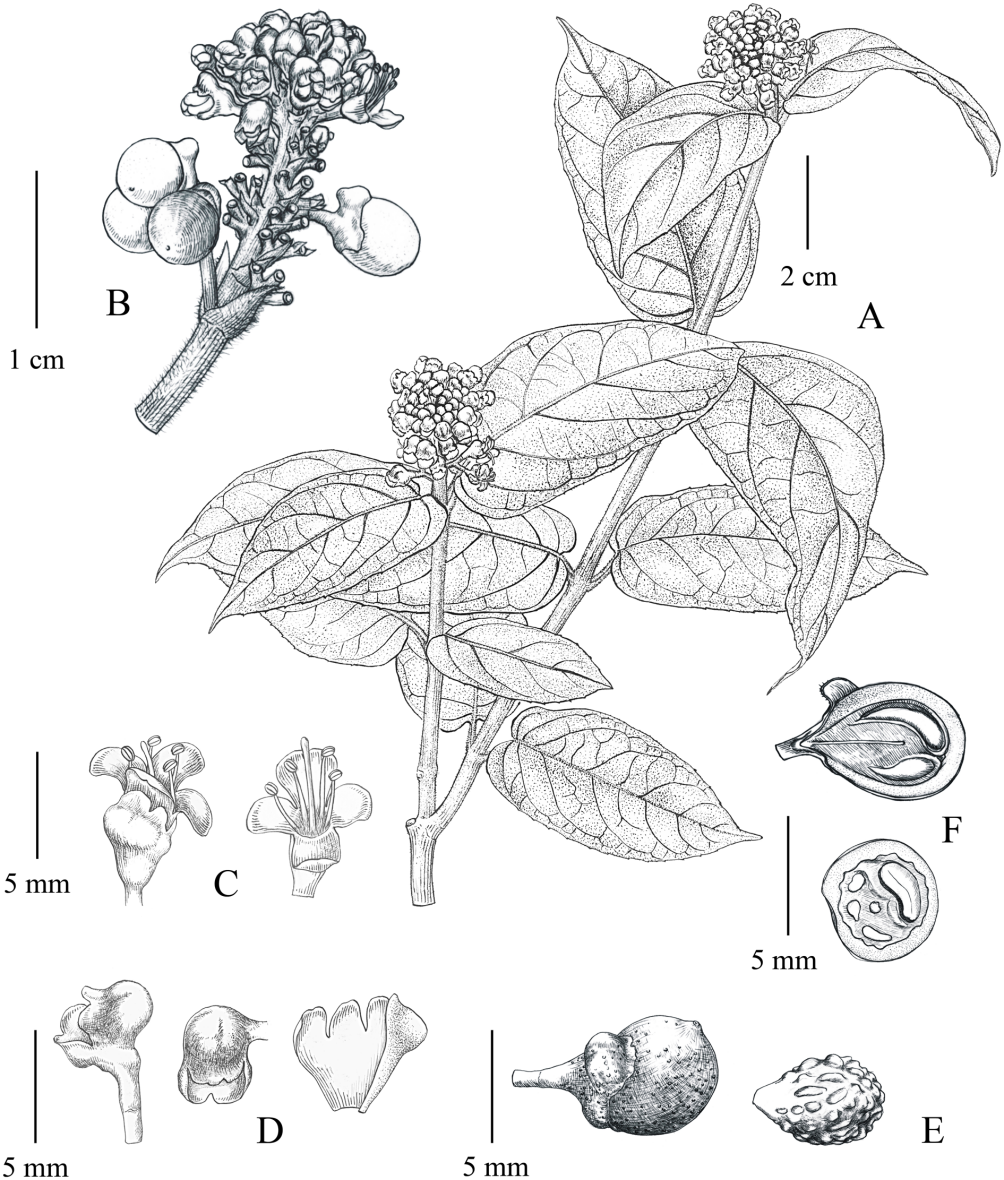
Illustration of *Premna vietnamensis*. **A**, A flowering branchlet. **B**, An infructescence. **C**, Flowers (left: adaxial view; right: abaxial view). **D**, Calyces (left: lateral view; middle: apical view; right: inside view after dissection). **E**, A mature fruit (left) and its pyrene (right). **F**, Longitudinal section (upper) and cross section (lower) of fruits.

***Premna vietnamensis*** Bo Li, ***sp*. *nov***. [urn:lsid:ipni.org:names:77177680–1] (Figs 1A, 1B, 2A and 4–6)

**Type**:—VIETNAM. Gia Lai Province, K’Bang District, Dong Commune, 14°13′03.5′′N, 108°36′27.9′′E, alt. 650 m, along roadside No DT669 and edges of primary evergreen broad-leaved forest on sandstone mountains, 28 April 2017, *D*. *V*. *Hai & N*. *S*. *Khang HK 28042017–01* (fl. and fr.) (holotype IBSC; isotypes HN, IBSC, JXAU).

#### Diagnosis

*Premna vietnamensis* is characterized by its calyx tube bearing a semi-globose fleshy appendage, which has not been reported in *Premna* to date. It is morphologically and geographically most similar to *P*. *stenobotrys*, but differs from it by an indument of dense golden hairs on the branchlets, petioles, and peduncles; much shorter petioles (2.0–6.0 mm vs. 15–50 mm), and by its yellow-greenish corolla and exserted stamens.

#### Description

Woody climbers, up to 5 m in length. Branchlets, petioles, leaf blades and inflorescences densely covered with long, spreading, golden-brown hairs. Branches grey, terete, sparsely dusty tomentose, with an interpetiolar ridge between petioles. Branchlets reddish brown, spreading, terete, without bracts at the base. Leaves simple, opposite, almost distichous, or slightly decussate, ovate-oblong to elliptic, rarely cordate-ovate, papery, (6.5–) 9.0–14 (–15.5) × 3.5–5.5 cm, apex acute, base cordate, margin serrate in the distal half of the leaf lamina; veins 4–6 pairs, abaxially raised and adaxially obviously compressed, secondary veins curved and jointed near margin; petiole short, 2.0–6.0 mm long, slightly inflated. Inflorescences terminal, a congested thyrse, subglobose to pyramid-shaped, 1.5–3.0 cm long, 2.0–2.5 cm wide, but infructescence elongated to 2.5–4.5 cm, peduncle 1.0–2.5 cm long; bracts lanceolate, ca. 1.0 mm long, easily deciduous; bracteoles subulate, tiny. Calyx campanulate, 2.0–3.0 mm long, green when young but purplish to brownish black when mature, obviously 2-lipped; upper lip emarginate to irregularly 2–3 lobed, covering a semi-globose fleshy appendage behind the lobes, lower lip 2 lobed, lobes slightly fleshy, apex rounded. Corolla greenish yellow, 2-lipped, 4.0–4.5 mm long; tube externally glabrous, internally densely white villose around throat; upper lip 1-lobed, upward reflexed, entire, oblong-obovate, apex rounded; lower lip 3-lobed, middle lobe oblong-obovate, straight or slightly recurved, lateral lobes rounded to obovate, strongly backward reflexed. Stamens 4, subequal, filaments yellowish to greenish white, glabrous, upward reflexed, slightly exserted; anther white. Ovary globose, 1.0–1.5 mm long, glabrous; style yellowish to greenish white, slender, upward reflexed. Fruits drupaceous, clavoid to nearly globose, 5.0–6.0 × 4.5–5.5 mm; exocarp membranous, green when young, from yellowish green, purplish green to bluish brown when mature, sarcocarp succulent, endocarp hard, warty; one pyrene with four seeds, only one fully develops.

#### Etymology

The specific epithet of this new species, “*vietnamensis*”, is derived from the name of the country, Vietnam, where this distinct species was originally collected and has not been observed in other countries so far.

#### Phenology

Flower buds were observed in late February to early March. Flowering was observed from mid-March to early May and fruiting from late April to late May.

#### Distribution and habitat

**Fig 7:**
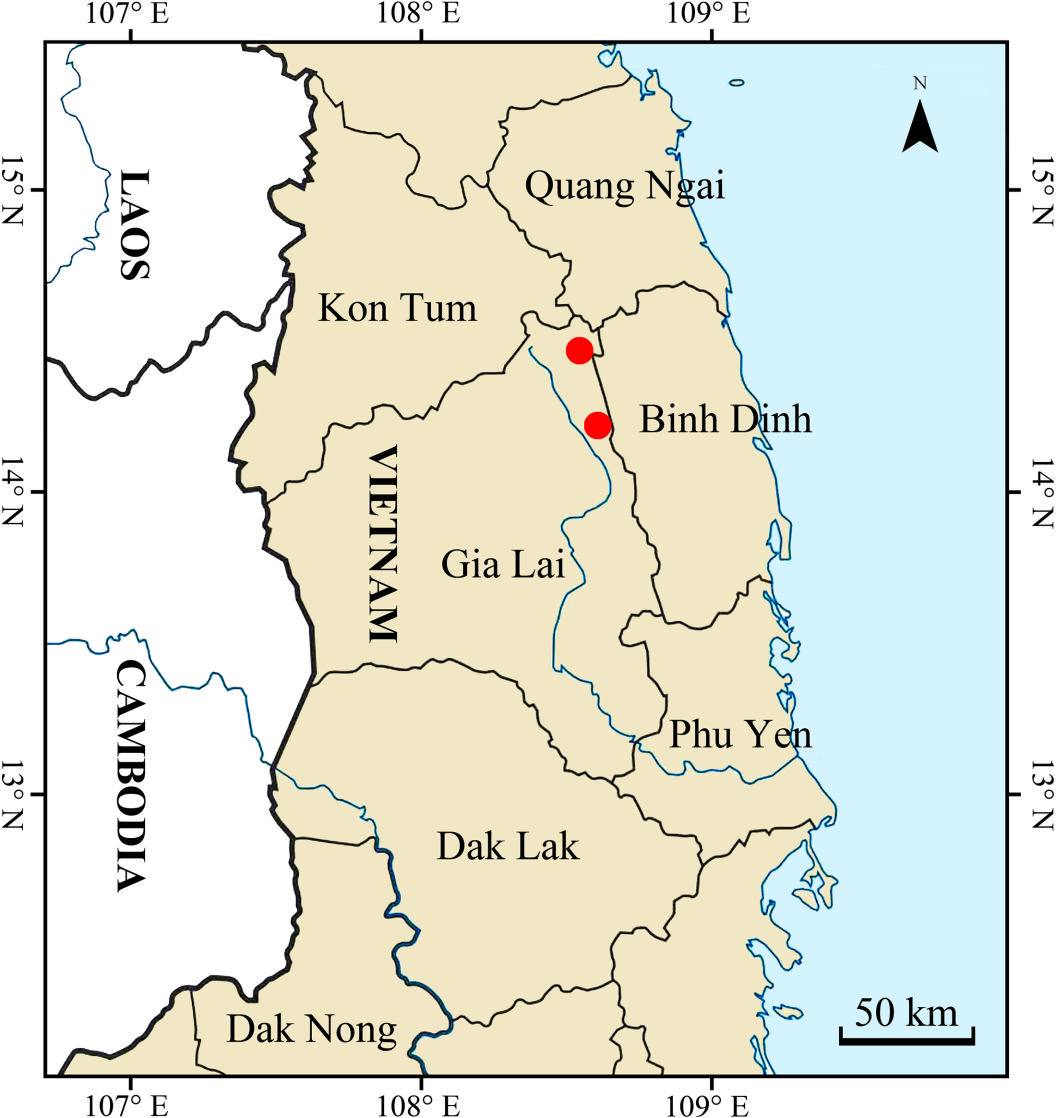
Distribution of *Premna vietnamensis* in Gia Lai Province of Vietnam.

*Premna vietnamensis* is currently known only from two localities in K’Bang District of Gia Lai Province, Central Highlands of Vietnam Vietnam: Dong Commune and Song Lang Commune, which are ca. 45 km apart (Fig 7). The plant grows well along roadside and edges of primary evergreen broad-leaved forest at elevations around 600–650 m. a.s.l., and climbs up to 5 m on many kinds of shrubs.

#### Additional specimens examined (paratypes)

VIETNAM. Gia Lai Province, K’Bang District, Dong Commune, 14°13′05.5′′N, 108°36′27.8′′E, alt. 645 m, along roadside No DT669 and edges of primary evergreen broad-leaved forest on sandstone mountains, 28 April 2017, *D*. *V*. *Hai & N*. *S*. *Khang HK 28042017–02* (fl. and fr.) (HN, IBSC, JXAU); Dong Commune, 14°12′16.3′′N, 108°36′17.8′′E, alt. 615 m, edges of primary evergreen broad-leaved forest on sandstone mountains, 28 April 2017, *D*. *V*. *Hai & N*. *S*. *Khang HK 28042017–03* (fl. and fr.) (HN, IBSC, JXAU); Dong Commune, 14°12′16′′N, 108°36′18′′E, 9 March 2011, *T*. *T*. *Bach et al*. *VK 4457* (fl.) (HN); Son Lang Commune, 25 April 1978, *T*. *D*. *Ly 586* (fl.) (HN); Son Lang Commune, 26 April 1978, *L*. *K*. *Bien 830* (HN); Son Lang Commune, 26 April 1978, *L*. *K*. *Bien 833* (fl.) (HN).

#### Conservation status

Based on our experiences on specimen examination and field observation, we consider *P*. *vietnamensis* as a rare species endemic to Gia Lai Province of Vietnam. It has been observed only in two separate sites in K’Bang District, although in this province it is very common to find similar habitats. Due to insufficient field surveys so far, we have not outlined the precise distribution boundaries of the species, and not ascertained its population status. Therefore, the information currently obtained is too inadequate to assess the species’ risk of extinction, whether directly or indirectly. In accordance with the IUCN Red List Categories [35], we propose to temporarily list the species as a taxon under the Data Deficient (DD) category. Further field surveys in Gia Lai Province of Vietnam are needed to gain more information on its abundance and/or distribution.

## Supporting information

S1 Table GenBank accession numbers and voucher information for the materials used in this study. (XLSX)
